# The combination of PF-429242 and chloroquine triggers pH-dependent cell death in hepatocellular carcinoma cells

**DOI:** 10.7150/ijms.109069

**Published:** 2025-05-10

**Authors:** Jiunn-Chang Lin, Tun-Sung Huang, Yan-Bin Chen, Pao-Shu Wu, Tsang-Pai Liu, Pei-Ming Yang

**Affiliations:** 1 Department of Surgery, MacKay Memorial Hospital, Taipei 10449, Taiwan; 2 MacKay Junior College of Medicine, Nursing, and Management, New Taipei City 11260, Taiwan; 3 Department of Medicine, MacKay Medical College, New Taipei City 25245, Taiwan; 4 Liver Medical Center, MacKay Memorial Hospital, Taipei 10449, Taiwan; 5 Ph.D. Program for Cancer Molecular Biology and Drug Discovery, College of Medical Science and Technology, Taipei Medical University, Taipei 11031, Taiwan; 6 Department of Pathology, MacKay Memorial Hospital, Taipei 10449, Taiwan; 7 Graduate Institute of Cancer Biology and Drug Discovery, College of Medical Science and Technology, Taipei Medical University, Taipei 11031, Taiwan; 8 TMU Research Center of Cancer Translational Medicine, Taipei 11031, Taiwan; 9 Cancer Center, Wan Fang Hospital, Taipei Medical University, Taipei 11696, Taiwan; 10 TMU and Affiliated Hospitals Pancreatic Cancer Groups, Taipei Medical University, Taipei 11031, Taiwan

**Keywords:** alkaliptosis, autophagy, hepatocellular carcinoma, pH-dependent cell death, regulated cell death

## Abstract

Hepatocellular carcinoma (HCC) remains a significant health challenge due to its resistance to conventional treatments and high recurrence rates. Developing novel therapeutic strategies is critical for improving outcomes for HCC patients. In this study, we identified the synergistic anticancer activity of the combination of PF-429242 and chloroquine against HCC cells. Combined treatment exhibited significant cytotoxicity against HCC cell lines, which was not observed with other therapeutic drugs. Notably, this synergistic effect was not mediated through apoptosis or autophagy. Further investigation revealed that the combination induced pH-dependent cell death, distinct from the previously described alkaliptosis. Unlike alkaliptosis, this cell death mechanism did not involve intracellular alkalinization or the IKKβ/NF-κB/CA9 signaling pathway. We also found that the ATP6V0D1/STAT3 axis, implicated in alkaliptosis, was not crucial for PF-429242/chloroquine-induced cell death. Additionally, site-1 protease inhibition by PF-429242 was not responsible for the observed synergistic effect. While the exact mechanism remains unclear, combined treatment induced a necrosis-like morphology and membrane rupture, which could be prevented by acidifying the culture medium. This research highlighted a novel pH-dependent cell death mechanism in HCC cells and suggests potential therapeutic implications for combining PF-429242 and chloroquine in cancer treatment.

## Introduction

Liver cancer represents a significant and escalating global health challenge. Among primary liver cancers, hepatocellular carcinoma (HCC) is the most common, accounting for 75%-85% of cases. Key risk factors contributing to HCC development include chronic infections with hepatitis B virus (HBV) or hepatitis C virus (HCV), as well as chronic liver conditions such as nonalcoholic fatty liver disease (NAFLD) and nonalcoholic steatohepatitis (NASH) 1. Standard treatment options for HCC encompass surgical resection, ablation, liver transplantation, and systemic pharmacological therapies. However, these options are only suitable for 15%-25% of patients and recurrent HCC occurs in over 70% of individuals following resection 2,3. In addition, HCC tends to be resistant to chemotherapy 4,5. New therapeutic options for advanced HCC include kinase and immune checkpoint inhibitors, but they only produce a temporary improvement in median overall survival 6,7. Therefore, novel therapeutic strategies are still urgently needed.

A significant clinical barrier to effective cancer treatment is the resistance of cancer cells to chemotherapy. Apoptosis initially contributes to the response of tumors to therapy, but cancer cells might later survive and develop apoptosis resistance 8. Thus, developing effective cancer therapy to overcome apoptosis resistance is urgently needed. New anticancer strategies may be developed by targeting non-apoptotic cell death, such as necroptosis, autophagy-dependent cell death, ferroptosis, and other regulated cell death (RCD) pathways 9. In particular, a newly identified form of RCD, known as alkaliptosis, which is pH-dependent, has emerged as a promising therapeutic strategy for targeting various types of cancer 10.

Our previous study showed that PF-429242, a site-1 protease inhibitor 11, exerts anticancer activity against HCC cells by inducing autophagic cell death 12. In this study, interestingly, the blockade of autophagic flux with chloroquine, a lysosomotropic agent 13-16, further enhanced the anticancer activity of PF-429242 by triggering pH-dependent cell death. Our results provide an innovative strategy for HCC therapy.

## Material and methods

### Chemicals and reagents

Minimum essential medium (MEM; #11095080), fetal bovine serum (FBS; #26140079), sodium pyruvate (#11360070), non-essential amino acids (NEAA; #11140050), alamarBlue cell viability reagent (#DAL1100), M-PER mammalian protein extraction reagent (#78501), pHrodo Green AM Intracellular pH Indicator (#P35373), Live Cell Imaging Solution (#A59688DJ), Intracellular pH Calibration Buffer Kit (#P35379), Lipofectamine 3000 (#L3000015), and RNAiMAX (#13778150) transfection reagents were purchased from Thermo Fisher Scientific. IκB (#GTX110521), phospho-Ser536-nuclear factor (NF)-κB/p65 (#GTX133899), ATP6V0D1 (GTX118317), STAT3 (#GTX104616), GFP (#GTX113617), and GAPDH (#GTX627408) antibodies were purchased form GeneTex. Poly ADP-ribose polymerase (PARP; #9542) and MAP1LC3B (LC3B; #2775) antibodies were purchased from Cell Signaling Technology. Caspase-3 (#ab13585) and carbonic anhydrase 9 (CA9; #ab184006) antibodies were purchased from Abcam. An anti-Flag antibody (#F3165) was purchased from Merck. Horseradish peroxidase (HRP)-labeled secondary antibodies were purchased from Jackson ImmunoResearch. Chloroquine (#C6628), N-acetylcysteine (NAC; #A7250), Trolox (#238813), ferrostatin-1 (#SML0583), deferoxamine (#D9533), cobalt chloride (#15862-1ML-F), and dimethyl sulfoxide (DMSO; #D5879) were purchased from Sigma. PF-429242 (#A11230), SC-514 (#A12746), IMD0354 (#A12837), MCC950 (#A13478), CA-074Me (#A13593), necrostatin-1 (#A11973), and Z-VAD-FMK (#A12373) were purchased from Adooq BioScience. Doxorubicin (#D-4000), sorafenib (#S-8502), regorafenib (#R-8024), and bafilomycin A1 (#B-1080) were purchased from LC Laboratory. JTC801 (#HY-13274) was purchased from MedChemExpress. A 7-aminoactinomycin D (7-AAD) viability staining solution (#402404) was purchased from BioLegend. A GENzol TriRNA kit (#GZX200) and RNase-free DNase I set (#DNS300) were purchased from Geneaid. IQ2 SYBR Green Fast qPCR System Master Mix (#DBU-006) was purchased from Bio-Genesis. An iScript cDNA synthesis kit (#1708891) was purchased from Bio-Rad Laboratories.

### Cell culture

The PLC/PRF/5 (PLC5; #60223) and HepG2 (#RM60025) cell lines were purchased from the Bioresource Collection and Research Center (BCRC; Hsinchu City, Taiwan). Cells were maintained in MEM supplemented with 0.1 mM NEAA, 1.0 mM sodium pyruvate, and 10% FBS. Cultures were incubated at 37°C in a humidified atmosphere with 5% CO₂.

### Cell viability assay

Assessment of cell viability was conducted using the alamarBlue cell viability reagent, a solution based on resazurin. Resazurin is a safe, cell-permeable compound initially blue in color that exhibits minimal fluorescence. Upon entry into living cells, it undergoes reduction to resorufin, thereby acquiring a red coloration and significant fluorescence. In this study, alterations in fluorescence, with excitation at 530-560 nm and emission at 590 nm, were employed to ascertain cell viability. The combined effect of drugs was calculated with Combenefit software 17.

### 7-AAD Staining Assay

The integrity of plasma membranes was assessed by determining the ability of cells to exclude 7-AAD, a membrane-impermeable, fluorescent DNA probe. 7-AAD/DNA complexes can be excited by a 488-nm laser and has an emission maximum of 647 nm. After treatment, cells were stained with 1 μg/ml 7-AAD for 15 min in the dark. Levels of 7-AAD staining were observed under a fluorescence microscope.

### pH measurement

Extracellular pH values in culture media were measured with a pH meter. Intracellular pH values were assessed with the pHrodo Green AM Intracellular pH Indicator following the manufacturer's instructions. In brief, cells were plated onto a 96-well plate with a black wall and a clear bottom. After drug treatment, cells were rinsed with Live Cell Imaging Solution (LCIS) and subsequently loaded with the pH indicator within LCIS. Following a 30-min incubation at 37 °C, the fluorescence was measured by excitation at 509 nm and emission at 533 nm. Intracellular pH values were determined using the Intracellular pH Calibration Buffer Kit.

### Transient Transfection

Transient plasmid overexpression (forward transfection) and small interfering (si)RNA knockdown (reverse transfection) analyses were respectively performed using Lipofectamine 3000 and RNAiMAX transfection reagents according to the manufacturer's instructions. After transfection for 24-48 h, cells were used for further experiments. siRNA specific for the *LC3B* (#sc-43390), *ATP6V0D1* (#sc-63207), *MBTPS1* (#sc-36496), and *MBTPS2* (#sc-41652) genes were purchased from Santa Cruz Technology. pEGFP-STAT3 (#111934), pcDNA3-SREBP1a (#26801), pcDNA3-SREBP1c (#26802), and pcDNA3-SREBP2 (#26807) plasmids were purchased from Addgene. The pCMV3-CA9 (#HG10107-UT) plasmid was purchased from Sino Biological.

### Real-time Quantitative Polymerase Chain Reaction (qPCR)

Total RNA was extracted using a GENzol TriRNA kit, following the manufacturer's instructions. First-strand cDNA synthesis was performed with an iScript cDNA synthesis kit, and a qPCR was carried out using IQ2 SYBR Green Fast qPCR System Master Mix on a LightCycler 96 System (Roche, Indianapolis, IN, USA) in triplicate. Primer sequences for amplification included *MBTPS1* (forward: 5′-TCCAATTGCTTGGATGACAG-3′; reverse: 5′-TCCAGAACCTTGGAGTACCG-3′), *MBTPS2* (forward: 5′-ACCCGTCAATCAACTGACCT-3′; reverse: 5′-TGCCAGATACCTGCACAAAA-3′), and *18S ribosomal (r)RNA* (forward: 5′-CGGCGACGACCCATTCGAAC-3′; reverse: 5′-GAATCGAACCCTGATTCCCCGTC-3′). No-reverse transcription controls were included to ensure the absence of contamination. Gene expression levels were quantified using the comparative CT (ΔΔCT) method, with *18S rRNA* as the reference gene and untreated cells as the calibrator.

### Western Blotting

Total cellular proteins were extracted using the M-PER Mammalian Protein Extraction Reagent, followed by a 30-min incubation on ice. Lysates were centrifuged at 13,000 ×*g* for 30 min at 4 °C, and the supernatant was collected. After determining protein concentrations using the Bio-Rad Protein Assay, equal amounts (50 μg) of protein were resolved on a 7%-12% sodium dodecyl sulfate (SDS)-polyacrylamide gel and transferred to a nitrocellulose membrane. The membrane was incubated with the appropriate primary antibody overnight at 4°C, washed thoroughly, and then treated with a HRP-conjugated secondary antibody for 30 min at room temperature. Immunoblots were visualized using an enhanced chemiluminescence (ECL) reagent.

### Statistical analysis

All data are presented as the mean ± standard deviation (SD), calculated from at least three independent experiments. Statistical analysis was performed using a two-tailed paired Student's *t*-test, with *p* < 0.05 considered indicative of statistical significance.

## Results

### Combined treatment with PF-429242 and chloroquine induces synergistic anticancer activity

**Figure 1:**
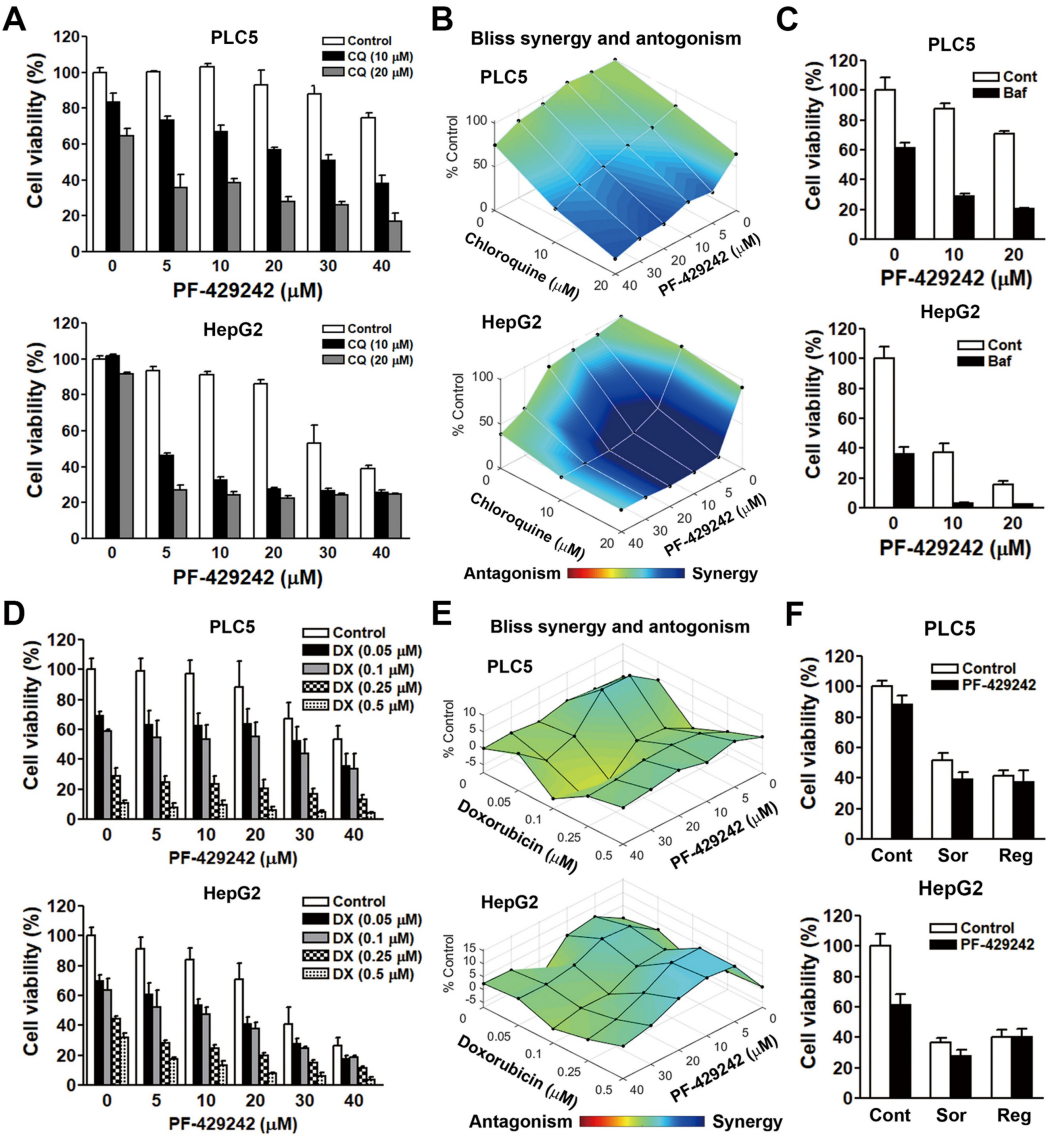
** Synergistic anticancer effect of the combination of PF-429242 and chloroquine (CQ). (A, B)** PLC5 and HepG2 cells were exposed to various concentrations of PF-429242, CQ, and their combination for 72 h. Cell viability was assessed using alamarBlue staining (A), and the synergistic effects of the drug combination were evaluated with Combenefit software applying the Bliss model (B). **(C)** PLC5 and HepG2 cells were treated with PF-429242, either alone or in combination with 1 nM bafilomycin A1 (Baf), for 72 h. Cell viability was analyzed using alamarBlue staining. **(D, E)** Cells were subjected to multiple doses of PF-429242, doxorubicin (DX), and their combinations over 72 h. Cell viability was measured with alamarBlue staining (D), and the Bliss method in Combenefit software was use to quantify drug synergy (E). **(F)** Treatment of PLC5 and HepG2 cells with 20 µM PF-429242, either alone or in combination with 5 µM sorafenib (Sor) or 10 µM regorafenib (Reg), was conducted for 72 h. Cell viability was evaluated using alamarBlue staining.

Chloroquine is a lysosomotropic agent that readily crosses the lysosomal membrane, becomes protonated, and accumulates within the lysosome, resulting in an increase in the lysosomal pH, which then inactivates acid hydrolase enzymes 18,19. Chloroquine is commonly used to block autophagic flux. Our previous study identified that PF-429242 induced autophagic cell death in HCC cells 12. We incidentally found that a combination of PF-429242 and chloroquine exhibited synergistic anticancer activity against two HCC cell lines: PLC/PRF/5 (hereafter denoted as PLC5) and HepG2 cells (**Figure 1A**) The synergism between PF-429242 and chloroquine was calculated with Combenefit software 17 using the Bliss method (**Figure 1B**). To confirm the effect of chloroquine, another lysosomotropic agent (bafilomycin A1) was used. Bafilomycin A1 can either inhibit vacuolar-type H+-ATPase (V-ATPase) that acidifies the lysosome or disrupt calcium-dependent autophagosome-lysosome fusion 20. Like chloroquine, bafilomycin A1 also enhanced PF-429242- induced cytotoxicity (**Figure 1C**). To investigate whether PF-429242 also synergizes with other therapeutic drugs to kill HCC cells, doxorubicin was used to treat PLC5 and HepG2 cells in combination with PF-429242. However, no synergistic anticancer effect was found (**Figure 1D**, **E**). In addition, sorafenib and regorafenib, two molecular-targeted agents approved for HCC treatment 21,22, did not exhibit synergistic anticancer activity when combined with PF-429242 (**Figure 1F**).

### Apoptosis and autophagy are not involved in the synergistic anticancer activity of PF-429242 and chloroquine

**Figure 2:**
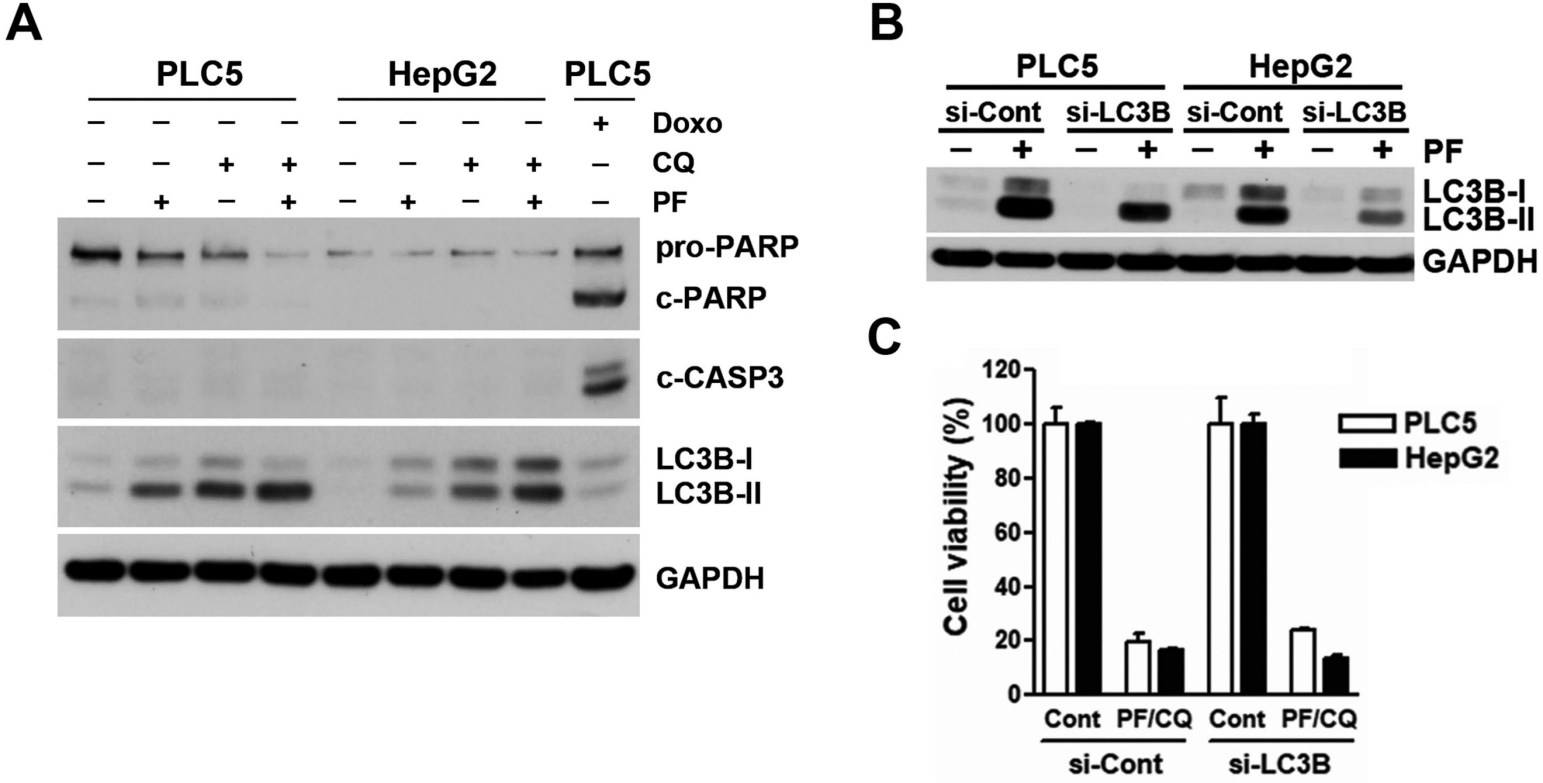
** Contribution of apoptosis and autophagy to PF-429242/chloroquine (CQ)-induced cell death. (A)** PLC5 and HepG2 cells were treated for 48 h with 10 µM PF-429242, 20 µM CQ, their combination (10 µM PF-429242 + 20 µM CQ), or 0.5 µM doxorubicin (DX, for PLC5 cells). Protein expression levels were analyzed via Western blotting. **(B)** PLC5 and HepG2 cells were transfected with LC3B siRNA for 24 h, followed by treatment with 20 µM PF-429242 for an additional 24 h. Western blotting was used to assess protein expression levels. **(C)** LC3B siRNA-transfected PLC5 and HepG2 cells were replated in 96-well plates and treated with a combination of 10 µM PF-429242 and 20 µM CQ (PF/CQ) for 72 h. Cell viability was evaluated using alamarBlue staining.

To investigate whether apoptosis was involved in the synergistic anticancer activity of PF-429242 and chloroquine, Western blotting was performed to analyze the level of apoptosis by the cleavages of PARP and caspase-3. Unlike doxorubicin (a positive control for inducing apoptosis), treatment with PF-429242, chloroquine, and their combinations did not induce apoptosis (**Figure 2A**), suggesting that other forms of cell death were responsible for the synergistic anticancer activity of PF-429242 and chloroquine. We also confirmed the induction of autophagy by PF-429242 as indicated by the accumulation of LC3B-II (**Figure 2A**). Chloroquine enhanced PF-429242-induced LC3B-II accumulation (**Figure 2A**), validating the blockade of autophagic flux. To ascertain the role of autophagy in the synergistic anticancer activity of PF-429242 and chloroquine, endogenous LC3B expression and PF-429242-induced LC3B-II accumulation were knocked down by siRNA (**Figure 2B**). However, LC3B-knockdown did not affect the cell viability in HCC cells treated with PF-429242 and chloroquine (**Figure 2C**). Therefore, neither apoptosis nor autophagy was involved in the synergistic anticancer activity of PF-429242 and chloroquine.

### Combined treatment with PF-429242 and chloroquine induces pH-dependent cell death

**Figure 3:**
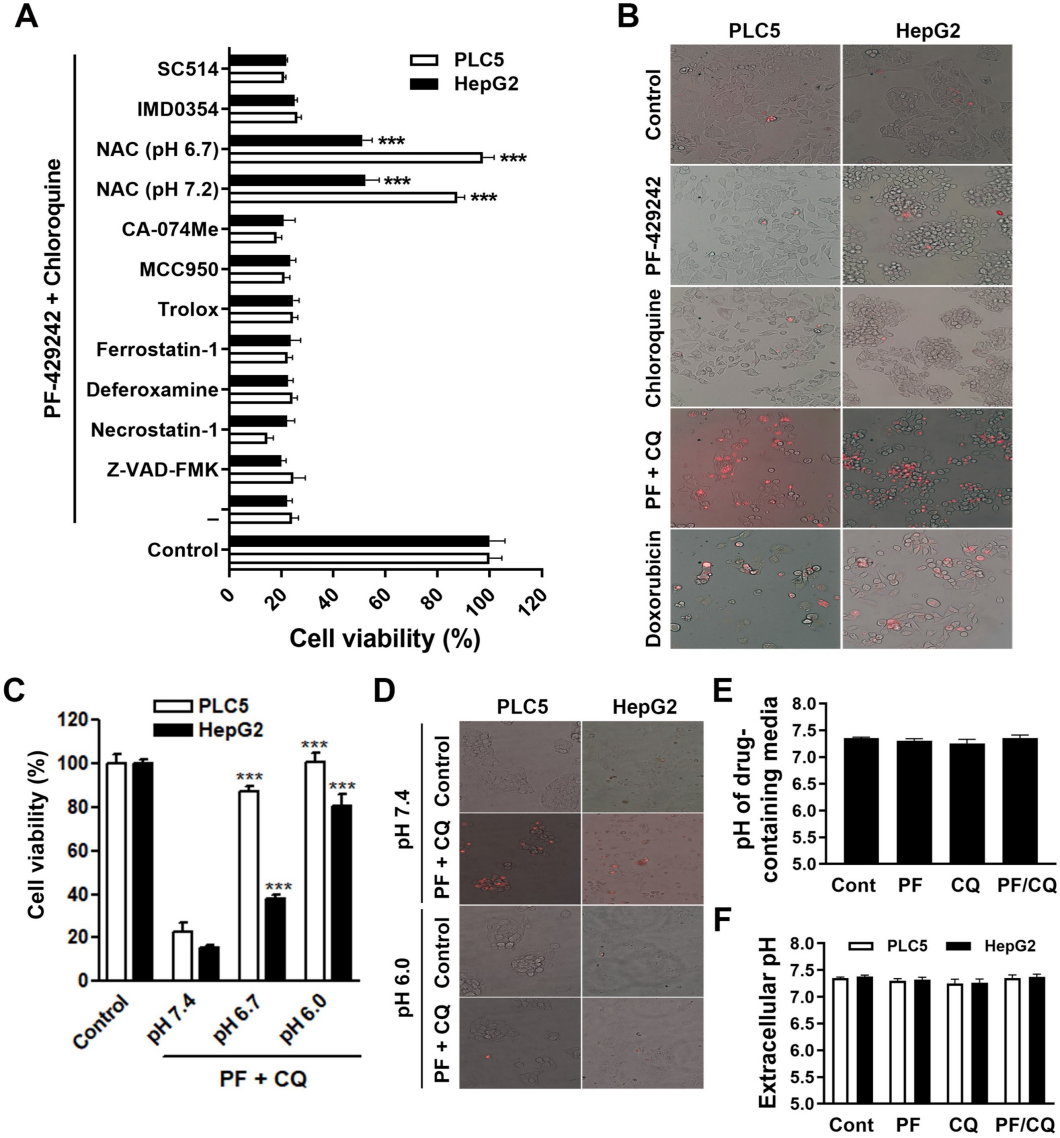
** pH-dependent cell death induced by the combination of PF-429242 and chloroquine. (A)** PLC5 and HepG2 cells were treated with 10 µM PF-429242 and 20 µM chloroquine (PF/CQ) for 72 h, with or without various inhibitors, antioxidants, or pH modulators. These included 20 µM Z-VAD-FMK, 5 µM necrostatin-1, 10 µM deferoxamine, 20 µM ferrostatin-1, 100 µM Trolox, 5 µM MCC950, 50 µM CA-074Me, 10 mM N-acetylcysteine (NAC), 1 µM IMD0354, and 50 µM SC514. Treatments were conducted under neutral (pH 7.2) and slightly acidic (pH 6.7) conditions. Cell viability was assessed via alamarBlue staining, with *** denoting a statistically significant reduction (*p* < 0.001) compared to PF/CQ-treated cells alone.** (B)** PLC5 and HepG2 cells were treated with 10 µM PF-429242, 20 µM chloroquine, their combination (PF/CQ), or 0.5 µM doxorubicin (DX, for PLC5 cells) for 48 h. The plasma membrane integrity was evaluated using 7-AAD staining, where cells with red fluorescence indicated 7-AAD positivity. Doxorubicin served as a control due to its induction of secondary necrosis during late-stage apoptosis. **(C)** PLC5 and HepG2 cells were exposed to 10 µM PF-429242 + 20 µM chloroquine for 72 h in culture media adjusted to pH 7.4, 6.8, or 6.0. Cell viability was measured using alamarBlue staining, with *** indicating a significant reduction (*p* < 0.001) in viability compared to cells treated at pH 7.4.** (D)** Plasma membrane integrity was further analyzed using 7-AAD staining following 48-h co-treatment with 10 µM PF-429242 and 20 µM chloroquine in PLC5 and HepG2 cells. **(E)** The pH of drug-containing culture media was measured with a pH meter to confirm the environmental conditions. **(F)** Extracellular pH levels in culture media were assessed using a pH meter after 24-h treatments with 10 µM PF-429242, 20 µM chloroquine, or their combination.

To systematically investigate the type of cell death responsible for PF-429242/chloroquine-induced synergistic anticancer activity, we employed various inhibitors of apoptosis (Z-VAD-FMK), necroptosis (necrostatin-1), ferroptosis (deferoxamine, ferrostatin-1, Trolox which is a water-soluble analog of vitamin E, and NAC), pyroptosis (MCC950), lysosome-dependent cell death (CA-074Me, deferoxamine, and NAC), alkaliptosis (NAC, IMD0354, and SC-514), and oxeiptosis (NAC) (as reviewed in 23). Among these inhibitors, NAC is a synthetic precursor of the endogenous cellular antioxidant glutathione (GSH). Because NAC is a weak acid, the cell culture medium became acidic (pH 6.7) when we prepared the working solution. We adjusted the pH to 7.2 and NAC in both neutral and acidic conditions was used to treat cells. As shown in **Figure 3A**, PLC5 and HepG2 cells were only rescued from PF-429242/chloroquine-induced cytotoxicity by acidic and neutral NAC. Other antioxidants, such as ferrostatin-1 and Trolox, did not exhibit a rescue effect, suggesting that the antioxidant activity of NAC was not involved in its cytoprotective effect. It was reported that the low pKa (3.252) of NAC results in time-dependent acidification of NAC-containing culture media 24. Therefore, it is possible that the combination of PF-429242 and chloroquine induces intracellular alkalinization and then cell death (alkaliptosis), which can be reversed by the pH-lowering effect of NAC. Surprisingly, IMD0354 and SC-514, two I-kappa-B-kinase beta (IKKβ) inhibitors that inhibit JTC801-induced alkaliptosis 25, failed to rescue cells from PF-429242/chloroquine-induced cytotoxicity (**Figure 3A**), indicating that one or more alternative mechanisms may be involved. Similar to JTC801-induced alkaliptosis 25, a combination of PF-429242 and chloroquine induced a necrosis-like morphology (plasma membrane rupture) as detected by the staining of 7-AAD, a plasma membrane-impermeable fluorescent DNA-binding dye (**Figure 3B**). To confirm that a combination of PF-429242 and chloroquine induced pH-dependent cell death, PLC5 and HepG2 cells were cultured in media adjusted to different pH values. As shown in **Figure 3C** and **3D**, the adjustment of the culture media's pH values from pH 7.4 to 6.0 almost fully prevented PF-429242/chloroquine-induced cytotoxicity and plasma membrane rupture. Furthermore, pH values of drug-containing media were not changed during preparation (**Figure 3E**) and drug treatment for 24 h (**Figure 3F**), indicating that intracellular, but not extracellular, pH fluctuations may have contributed to the pH-dependent cell death induced by combined PF-429242/chloroquine treatment. Therefore, the combination of PF-429242 and chloroquine triggered pH-dependent cell death.

### PF-429242/chloroquine-induced pH-dependent cell death is distinct from JTC801-induced alkaliptosis

**Figure 4:**
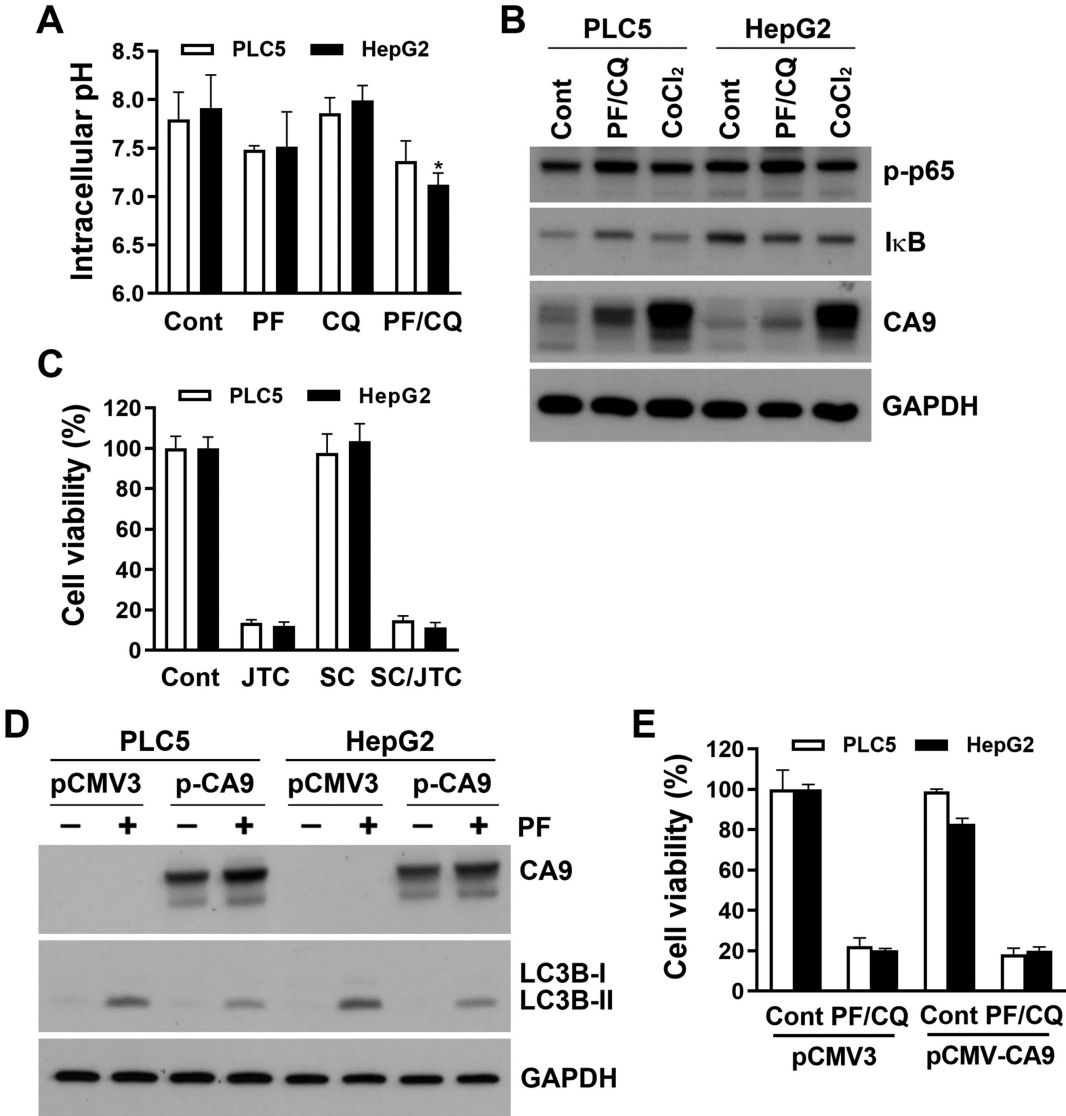
** Influence of intracellular pH and the NF-κB/CA9 pathway on PF-429242/chloroquine-induced cell death. (A)** PLC5 and HepG2 cells were treated with 10 µM PF-429242, 20 µM chloroquine, or their combination (PF/CQ) for 24 h. Intracellular pH levels were measured using the pHrodo™ Green AM Intracellular pH Indicator. * indicates a statistically significant difference (*p* < 0.05) compared to untreated control cells. **(B)** Cells were co-treated with 10 µM PF-429242 and 20 µM chloroquine or exposed to 100 µM cobalt chloride (CoCl₂) for 24 h. Protein expression levels were assessed via a Western blot analysis.** (C)** PLC5 and HepG2 cells were treated with JTC801 (1.5 µM for HepG2 and 3 µM for PLC5), 10 µM SC-514, or their combination for 72 h. Cell viability was evaluated using alamarBlue staining. **(D)** Cells were transfected with a CA9 plasmid for 24 h, followed by treatment with 20 µM PF-429242 for another 24 h. Protein expression levels were examined using Western blotting. **(E)** After CA9 transfection, PLC5 and HepG2 cells were replated in 96-well plates and co-treated with 10 µM PF-429242 and 20 µM chloroquine for 72 h. Cell viability was measured using alamarBlue staining.

Intracellular alkalinization by activation of IKKβ/NF-κB and the subsequent reduction of CA9 expression is the major route for JTC801-induced alkaliptosis 25. Because IKKβ inhibitors failed to rescue PF-429242/chloroquine-induced cell death, we further measured intracellular pH levels using a pHrodo™ Green AM Intracellular pH Indicator. Surprisingly, PF-429242 alone or in combination with chloroquine induced mild intracellular acidification, although it did not reach statistical significance, except for the PF-429242/chloroquine combination sample in HepG2 cells (**Figure 4A**). This suggests that intracellular alkalization was not implicated in PF-429242/chloroquine-induced pH-dependent cell death. We also explored whether the NF-κB/CA9 signaling pathway was altered by the combined treatment with PF-429242 and chloroquine. As shown in **Figure 4B**, PF-429242/chloroquine combined treatment did not activate NF-κB, as indicated by the levels of p65 phosphorylation and IκB expression. In addition, endogenous CA9 expression was very low in PLC5 and HepG2 cells, and PF-429242/chloroquine combined treatment slightly induced CA9 expression (**Figure 4B**). CA9 is known to be transcriptionally induced by hypoxia-inducible factor (HIF)-1 during hypoxia 26. As a positive control, chemical hypoxia by cobalt chloride (CoCl_2_) dramatically induced CA9 expression (**Figure 4B**). These results implied that PLC5 and HepG2 cells might not rely on endogenous CA9 for survival and thus were less susceptible to alkaliptosis. Indeed, JTC801-induced cytotoxicity was not reversed by SC-514 in PLC5 or HepG2 cells (**Figure 4C**). To further clarify CA9's role, PLC5 and HepG2 cells were transfected with a CA9-encoding plasmid. CA9 overexpression slightly attenuated PF-429242-induced autophagy (**Figure 4D**), but did not alter PF-429242/chloroquine-induced cytotoxicity (**Figure 4E**).

**Figure 5:**
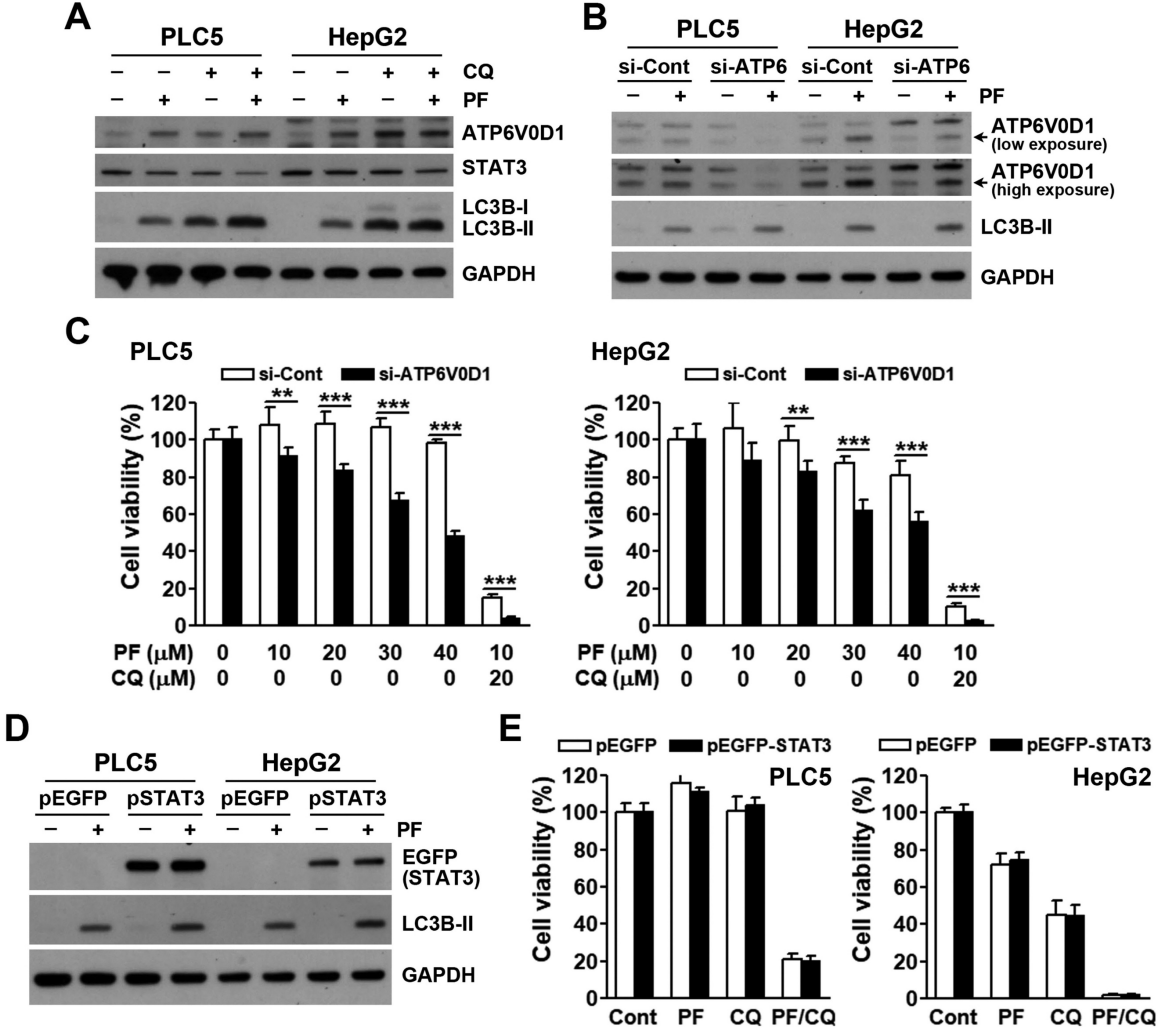
** Contribution of ATP6V0D1/STAT3 to PF-429242/chloroquine-induced cell death. (A)** PLC5 and HepG2 cells were exposed to 10 µM PF-429242, 20 µM chloroquine, or their combination (PF/CQ) for 24 h. Protein expression levels were analyzed via Western blotting. **(B)** PLC5 and HepG2 cells were transfected with ATP6V0D1 siRNA for 24 h, followed by treatment with 20 µM PF-429242 for an additional 24 h. Western blotting was used to assess protein expression levels.** (C)** ATP6V0D1 siRNA-transfected PLC5 and HepG2 cells were replated in 96-well plates and treated with PF-429242 and its combination with chloroquine (PF/CQ) for 72 h. Cell viability was evaluated using alamarBlue staining. **(D)** PLC5 and HepG2 cells were transfected with a STAT3 plasmid for 24 h, followed by a 24-h treatment with 20 µM PF-429242. Protein expression levels were assessed via Western blotting. **(E)** STAT3-transfected cells were replated into 96-well plates and co-treated with 10 µM PF-429242 and 20 µM chloroquine for 72 h. Cell viability was evaluated using alamarBlue staining.

Recently, JTC801 was found to directly bind and stabilize ATPase H^+^ transporting V0 subunit D1 (ATP6V0D1), a V-ATPase member. ATP6V0D1 then interacts and inhibits signal transducer and activator of transcription 3 (STAT3), leading to lysosomal pH dysfunction and alkaliptosis 27. To investigate the role of ATP6V0D1/STAT3 in PF-4294242/chloroquine-induced cell death, their protein levels were determined. As shown in **Figure 5A**, PF-429242 and chloroquine alone and/or additively inhibited STAT3 and induced ATP6V0D1 expression. However, ATP6V0D1-knockdown did not rescue, but enhanced, PF-429242- and PF-429242/chloroquine-induced cell death (**Figure 5B**, **C**). Furthermore, STAT3 overexpression had no effect on cell death induced by PF-429242 and chloroquine, alone or in combination (**Figure 5D**, **E**). Therefore, PF-429242/chloroquine combined treatment induced a form of pH-dependent cell death distinct from JTC801-induced alkaliptosis.

### Site-1 protease inhibition does not synergize with chloroquine to kill HCC cells

**Figure 6:**
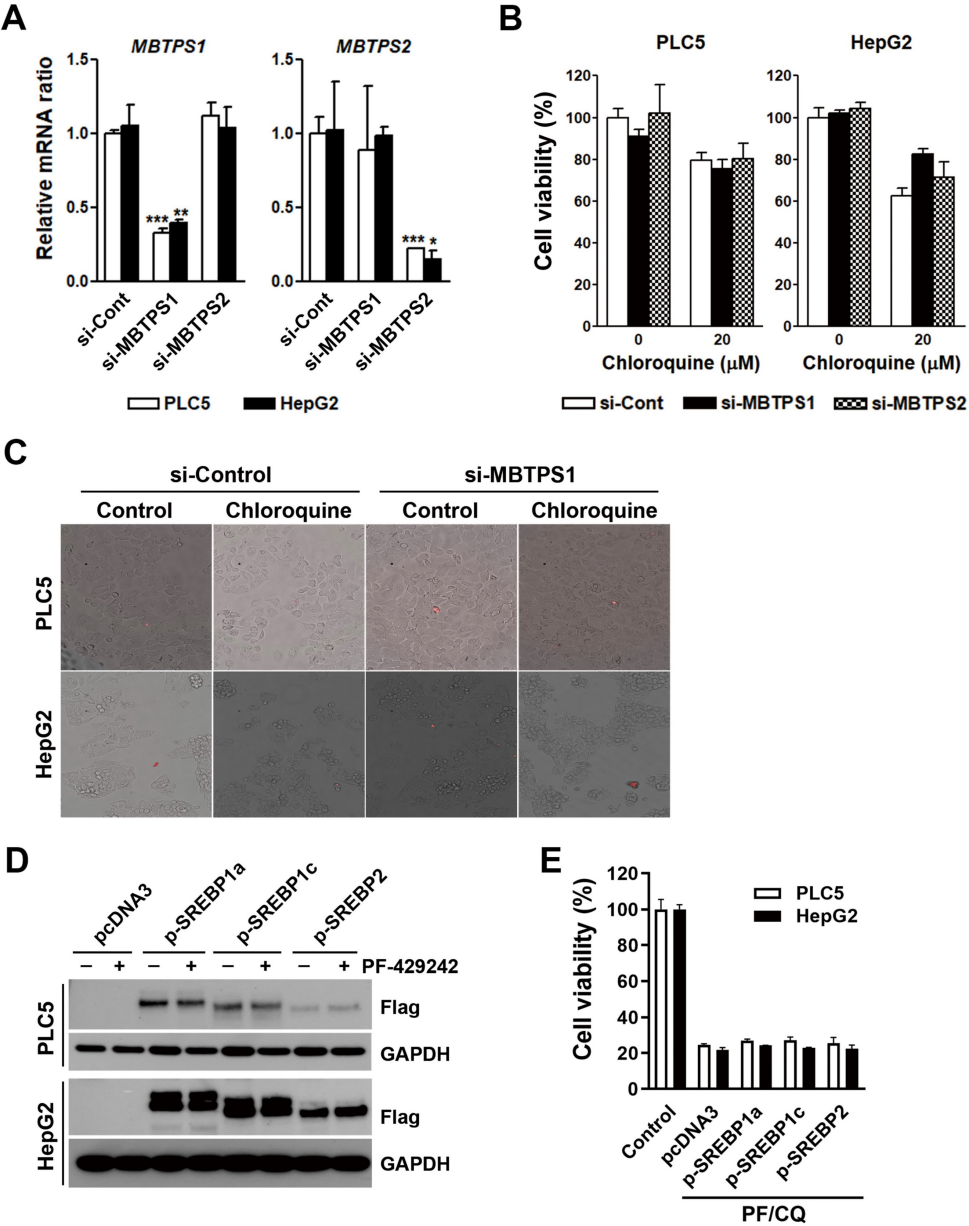
** Involvement of SREBP signaling in PF-429242/chloroquine-induced cell death. (A)** PLC5 and HepG2 cells were transfected with siRNAs targeting *MBTPS1* or *MBTPS2* for 48 h. Following transfection, mRNA levels of *MBTPS1* and *MBTPS2* were measured using a real-time qPCR. *, **, and *** represent statistically significant differences (*p* < 0.05, *p* < 0.01, and *p* < 0.001, respectively) compared to control siRNA-transfected cells. **(B)** Cells transfected with MBTPS1 or MBTPS2 siRNAs were replated into 96-well plates. After 72-h treatment with 20 µM chloroquine, cell viability was assessed using alamarBlue staining.** (C)** PLC5 and HepG2 cells transfected with *MBTPS1* or *MBTPS2* siRNAs for 24 h were subsequently treated with 20 µM chloroquine for 48 h. Plasma membrane integrity was evaluated using 7-AAD staining, with red fluorescence indicating 7-AAD-positive cells.** (D)** Cells were transfected with plasmids encoding SREBP1a/c or SREBP2 for 24 h and then treated with 20 µM PF-429242 for an additional 24 h. Protein expression levels were analyzed via Western blotting. **(E)** SREBP1a/c- or SREBP2-transfected PLC5 and HepG2 cells were replated into 96-well plates and co-treated with 10 µM PF-429242 and 20 µM chloroquine (PF/CQ) for 72 h. Cell viability was measured using alamarBlue staining.

Because PF-429242 is a site-1 protease inhibitor, we further investigated whether site-1 protease inhibition by PF-429242 was involved in PF-429242/chloroquine-induced cell death. *MBTPS1* (which encodes a site-1 protease) and *MBTPS2* (which encodes a site-2 protease and was used for comparison) messenger (m)RNA expressions in PLC5 and HepG2 cells were knocked down by siRNAs (**Figure 6A**). However, *MBTPS1*- and *MBTPS2*-knockdown did not synergize with chloroquine to kill HCC cells or cause plasma membrane rupture (**Figure 6B**, **C**). Site-1 and site-2 proteases cleave the pro-forms of SREBP1/2. Mature SREBP1/2 enter into nuclei and regulate genes related to sterol biosynthesis 28. PLC5 and HepG2 cells were transfected with plasmids encoding SREBP1a/c and SREBP2 (**Figure 6D**). Consistently, SREBP1/2 overexpression did not rescue PF-429242/chloroquine-induced cytotoxicity (**Figure 6E**). Therefore, site-1 protease inhibition is not involved in PF-429242/chloroquine-induced cell death.

## Discussion

Results of this study unveil a novel approach for treating HCC by demonstrating the synergistic anticancer effects of PF-429242 and chloroquine. The combination of these drugs led to significant cytotoxicity against HCC cell lines, a phenomenon not observed with other therapeutic drugs such as doxorubicin, sorafenib, or regorafenib. This indicates a unique mechanism of action for the PF-429242/chloroquine combination, differentiating it from standard treatments. Importantly, the synergy was confirmed using another lysosomotropic agent, bafilomycin A1, further supporting the potential of this drug combination to induce potent anticancer effects by targeting lysosomal functions.

Chloroquine is widely used as an autophagy inhibitor and is often employed in clinical settings to counteract drug resistance resulting from autophagy induction in cancer cells 29. In our study, we specifically examined whether autophagy played a role in the observed cell death mechanism. Our findings indicate that the synergistic cytotoxicity of PF-429242 and chloroquine was not dependent on inhibition of autophagy. Instead, the combined treatment induced a distinct pH-dependent cell death mechanism. This suggests that our study effectively bypassed the conventional autophagy-mediated drug resistance pathway and instead leveraged a novel, alternative cell death mechanism that could be exploited for therapeutic benefit.

Our findings suggest that the synergistic effect of PF-429242 and chloroquine was not mediated through apoptosis or autophagy. This divergence from traditional cell death pathways prompted an investigation into alternative mechanisms. The observed pH-dependent cell death mechanism was notably distinct from known forms like alkaliptosis, as it did not involve intracellular alkalinization or the IKKβ/NF-κB/CA9 or ATP6V0D1/STAT3 pathways. Additionally, the role of site-1 protease inhibition by PF-429242 was ruled out, as neither knockdown of site-1 protease nor overexpression of SREBP1/2 affected the cytotoxicity induced by the drug combination. Further investigations are needed to elucidate the exact molecular mechanisms.

Lysosomal function is regulated by its internal pH, which is maintained in the range of pH 4.5-5 by V-ATPases and counter-ion transporters. V-ATPases are ATP-driven, pH-regulated proton pumps that acidify the lysosome. Due to the electrogenic nature of V-ATPases, parallel ion conductance is required for significant lysosomal acidification 30. An imbalanced lysosomal pH and lysosomal alkalization may inhibit lysosomal enzymes and impair the degradation capacity of lysosomes 31. Chloroquine and bafilomycin A1 can cause lysosomal alkalinization 32, which may contribute to their ability to synergize with PF-429242. Supporting this, ATP6V0D1-knockdown also enhanced the anticancer activity of PF-429242.

The discovery of a novel pH-dependent cell death mechanism presents significant implications for HCC treatment. Given the challenges associated with chemoresistance in HCC, targeting alternative forms of cell death represents a promising therapeutic strategy. Our findings suggest that lysosomal alkalinization, modulated by chloroquine, may play a key role in sensitizing HCC cells to PF-429242. This could open up avenues for repurposing chloroquine as a chemosensitizing agent in combination therapies for HCC. Additionally, pH modulation strategies could be further explored in conjunction with other drugs that disrupt lysosomal homeostasis.

While our study focused on HCC cells, we acknowledge that a comparison with normal hepatocytes would strengthen the precision and translational relevance of our findings. Future studies should incorporate non-cancerous hepatocyte models to assess whether the pH-dependent cell death mechanism is selectively cytotoxic to cancer cells. This could provide insights into the therapeutic window of PF-429242 and chloroquine combination treatment.

In conclusion, our study reveals a novel pH-dependent cell death mechanism induced by the combination of PF-429242 and chloroquine in HCC cells. This synergistic anticancer effect is distinct from traditional apoptosis, autophagy, and other known forms of cell death such as alkaliptosis. The findings suggest potential therapeutic implications for this drug combination, although further research is necessary to elucidate the precise molecular mechanisms involved. The unique pathway identified in this study offers a promising direction for developing targeted treatments for HCC.

## References

[1] Philips CA, Rajesh S, Nair DC (2021). Hepatocellular Carcinoma in 2021: An Exhaustive Update. Cureus.

[2] Tabrizian P, Jibara G, Shrager B (2015). Recurrence of hepatocellular cancer after resection: patterns, treatments, and prognosis. Ann Surg.

[3] Roberts LR, Gores GJ (2005). Hepatocellular carcinoma: molecular pathways and new therapeutic targets. Semin Liver Dis.

[4] Bray F, Ferlay J, Soerjomataram I (2018). Global cancer statistics 2018: GLOBOCAN estimates of incidence and mortality worldwide for 36 cancers in 185 countries. CA: a cancer journal for clinicians.

[5] Yang JD, Hainaut P, Gores GJ (2019). A global view of hepatocellular carcinoma: trends, risk, prevention and management. Nat Rev Gastroenterol Hepatol.

[6] Liu HT, Jiang MJ, Deng ZJ (2021). Immune Checkpoint Inhibitors in Hepatocellular Carcinoma: Current Progresses and Challenges. Front Oncol.

[7] Rinaldi L, Vetrano E, Rinaldi B (2021). HCC and Molecular Targeting Therapies: Back to the Future. Biomedicines.

[8] Fisher DE (1994). Apoptosis in Cancer-Therapy - Crossing the Threshold. Cell.

[9] Galluzzi L, Vitale I, Aaronson SA (2018). Molecular mechanisms of cell death: recommendations of the Nomenclature Committee on Cell Death 2018. Cell Death Differ.

[10] Liu J, Kuang F, Kang R (2020). Alkaliptosis: a new weapon for cancer therapy. Cancer Gene Ther.

[11] Hay BA, Abrams B, Zumbrunn AY (2007). Aminopyrrolidineamide inhibitors of site-1 protease. Bioorg Med Chem Lett.

[12] Lin JC, Liu TP, Chen YB (2023). PF-429242 exhibits anticancer activity in hepatocellular carcinoma cells via FOXO1-dependent autophagic cell death and IGFBP1-dependent anti-survival signaling. Am J Cancer Res.

[13] Abraham R, Hendy RJ (1970). Irreversible lysosomal damage induced by chloroquine in the retinae of pigmented and albino rats. Exp Mol Pathol.

[14] Ascoli M, Puett D (1978). Inhibition of the degradation of receptor-bound human choriogonadotropin by lysosomotropic agents, protease inhibitors, and metabolic inhibitors. J Biol Chem.

[15] Lie SO, Schofield B (1973). Inactivation of lysosomal function in normal cultured human fibroblasts by chloroquine. Biochem Pharmacol.

[16] Wibo M, Poole B (1974). Protein degradation in cultured cells. II. The uptake of chloroquine by rat fibroblasts and the inhibition of cellular protein degradation and cathepsin B1. J Cell Biol.

[17] Di Veroli GY, Fornari C, Wang D (2016). Combenefit: an interactive platform for the analysis and visualization of drug combinations. Bioinformatics.

[18] Schrezenmeier E, Dorner T (2020). Mechanisms of action of hydroxychloroquine and chloroquine: implications for rheumatology. Nat Rev Rheumatol.

[19] Solitro AR, MacKeigan JP (2016). Leaving the lysosome behind: novel developments in autophagy inhibition. Future Med Chem.

[20] Mauvezin C, Neufeld TP (2015). Bafilomycin A1 disrupts autophagic flux by inhibiting both V-ATPase-dependent acidification and Ca-P60A/SERCA-dependent autophagosome-lysosome fusion. Autophagy.

[21] Bruix J, Qin S, Merle P (2017). Regorafenib for patients with hepatocellular carcinoma who progressed on sorafenib treatment (RESORCE): a randomised, double-blind, placebo-controlled, phase 3 trial. Lancet.

[22] Llovet JM, Ricci S, Mazzaferro V (2008). Sorafenib in advanced hepatocellular carcinoma. N Engl J Med.

[23] Tang D, Kang R, Berghe TV (2019). The molecular machinery of regulated cell death. Cell Res.

[24] Lee YJ, Lee DM, Lee SH (2013). Production of Cyr61 protein is modulated by extracellular acidification and PI3K/Akt signaling in prostate carcinoma PC-3 cells. Food Chem Toxicol.

[25] Song X, Zhu S, Xie Y (2018). JTC801 Induces pH-dependent Death Specifically in Cancer Cells and Slows Growth of Tumors in Mice. Gastroenterology.

[26] van den Beucken T, Koritzinsky M, Niessen H (2009). Hypoxia-induced expression of carbonic anhydrase 9 is dependent on the unfolded protein response. J Biol Chem.

[27] Chen F, Zhu S, Kang R (2023). ATP6V0D1 promotes alkaliptosis by blocking STAT3-mediated lysosomal pH homeostasis. Cell Rep.

[28] Shimano H, Sato R (2017). SREBP-regulated lipid metabolism: convergent physiology - divergent pathophysiology. Nat Rev Endocrinol.

[29] Qin Y, Ashrafizadeh M, Mongiardini V (2023). Autophagy and cancer drug resistance in dialogue: Pre-clinical and clinical evidence. Cancer Lett.

[30] Zhang W, Bai J, Hang K (2022). Role of Lysosomal Acidification Dysfunction in Mesenchymal Stem Cell Senescence. Front Cell Dev Biol.

[31] Chen F, Kang R, Liu J (2022). The V-ATPases in cancer and cell death. Cancer Gene Ther.

[32] Fedele AO, Proud CG (2020). Chloroquine and bafilomycin A mimic lysosomal storage disorders and impair mTORC1 signalling. Biosci Rep.

